# The correlation between rapid eye movement sleep and nocturnal hypertension in patients with obstructive sleep apnea: A retrospective study

**DOI:** 10.1097/MD.0000000000040740

**Published:** 2024-11-29

**Authors:** Wenjing Zhang, Chunlin Tu, Yanfang Yu

**Affiliations:** a Department of Respiratory and Critical Care Medicine, Jiading District Central Hospital Affiliated Shanghai University of Medicine & Health Sciences, Shanghai, China.

**Keywords:** nocturnal hypertension, obstructive sleep apnea, rapid eye movement sleep

## Abstract

Obstructive sleep apnea (OSA) is a respiratory disease closely associated with hypertension and heart disease. This study aimed to evaluate the diagnostic value of rapid eye movement (REM) sleep during nocturnal hypertension in OSA patients. We selected 194 patients who underwent polysomnography (PSG) at the Respiratory and Critical Care Medicine Department of Jiading District Central Hospital in Shanghai between January 2021 and August 2023. All patients were assigned to the hypertension and normal blood pressure groups, and the differences between the 2 groups were compared. This study included 194 patients (137 with nocturnal hypertension and 57 with normal nocturnal blood pressure). The proportion of male sex, body mass index, apnea–hypopnea index (AHI), REM sleep duration, percentage of REM sleep duration to total sleep duration, REM-AHI, nonrapid eye movement-AHI, oxygen desaturation index (ODI), REM-ODI, nonrapid eye movement-ODI, and average nighttime heart rate during sleep were higher in the hypertension group than in the normal blood pressure group, and the lowest oxygen saturation was lower than that in the normal blood pressure group (*P* < .05). Logistic regression analysis showed that REM-AHI was an independent risk factor for nocturnal hypertension (adjusted odds ratio, 1.048; 95% confidence interval, 1.011–1.086; *P* = .01). Receiver operating characteristic curve analysis showed that the REM-AHI had an area under the curve of 0.721 (95% confidence interval, 0.641–0.801; *P* < .001) for diagnosing nocturnal hypertension in patients with OSA, with a maximum Youden index of 0.379. The optimal critical value of the REM-AHI was 23.6 times/h, with a sensitivity of 64.2% and specificity of 73.7%. REM sleep is closely related to nocturnal hypertension, and patients with OSA are more prone to cardiovascular events.

## 1. Introduction

Obstructive sleep apnea (OSA) is the most common type of sleep apnea–hypopnea.^[1]^ It mainly refers to repeated apnea and hypopnea caused by collapse and obstruction of the upper respiratory tract (pharynx) during sleep, ultimately leading to chronic intermittent hypoxia and sleep interruption. The typical symptoms include snoring and excessive drowsiness. The prevalence of OSA is 2 to 4%,^[2]^ and obesity is the main risk factor. In recent years, the prevalence of OSA has increased owing to increasing obesity and is estimated to affect up to 1 billion people worldwide.^[3]^

Human sleep can be roughly divided into 2 stages: rapid eye movement (REM) and nonrapid eye movement (NREM). REM sleep accounts for one-quarter of the sleep cycle.^[4]^ During REM sleep, the genioglossus muscle is more relaxed and airway collapse is more pronounced than during NREM sleep.^[5]^ This leads to a more frequent, longer-duration, and greater decrease in oxygen saturation during REM sleep.^[6]^ Under physiological conditions, blood pressure fluctuates throughout the day, with high blood pressure during the day, decreased blood pressure during sleep at night, and increased sympathetic nerve excitability considered one of the reasons for the increase in nocturnal blood pressure.^[7]^ Hypoxia induces increased sympathetic excitability, heart rate, and nocturnal blood pressure during REM sleep in patients with OSA. However, the effect of REM phase parameters on nocturnal hypertension in OSA patients remains unclear. This study aimed to evaluate the impact and diagnostic value of REM sleep in nocturnal hypertension in patients with OSA.

## 2. Materials and methods

### 2.1. Study subjects

In this retrospective study, 305 snoring patients who visited the Respiratory and Critical Care Medicine Department of Jiading District Central Hospital in Shanghai between January 2021 and August 2023 were selected. All the patients underwent polysomnography (PSG) and nocturnal ambulatory blood pressure monitoring. Based on the inclusion and exclusion criteria, 194 patients with OSA were included in the study. This study was approved by the Research Ethics Committee of the Jiading District Central Hospital in Shanghai, with 2 reviewers.

### 2.2. Inclusion criteria

The inclusion criteria were as follows: (1) age ≥ 18 years, (2) had completed ≥7 hours of PSG and ambulatory blood pressure monitoring, (3) had not received antihypertensive treatment in the past, and (4) did not undergo OSA-related treatment.

### 2.3. Exclusion criteria

The exclusion criteria were as follows: (1) central or mixed sleep apnea and hypopnea as the main causes; (2) concomitant secondary hypertension; (3) severe heart and lung disease, and other severe disease; (4) severe mental illness; (5) use of oral psychotropic drugs; (6) toxic intake.

### 2.4. Research process

**Figure 1.**
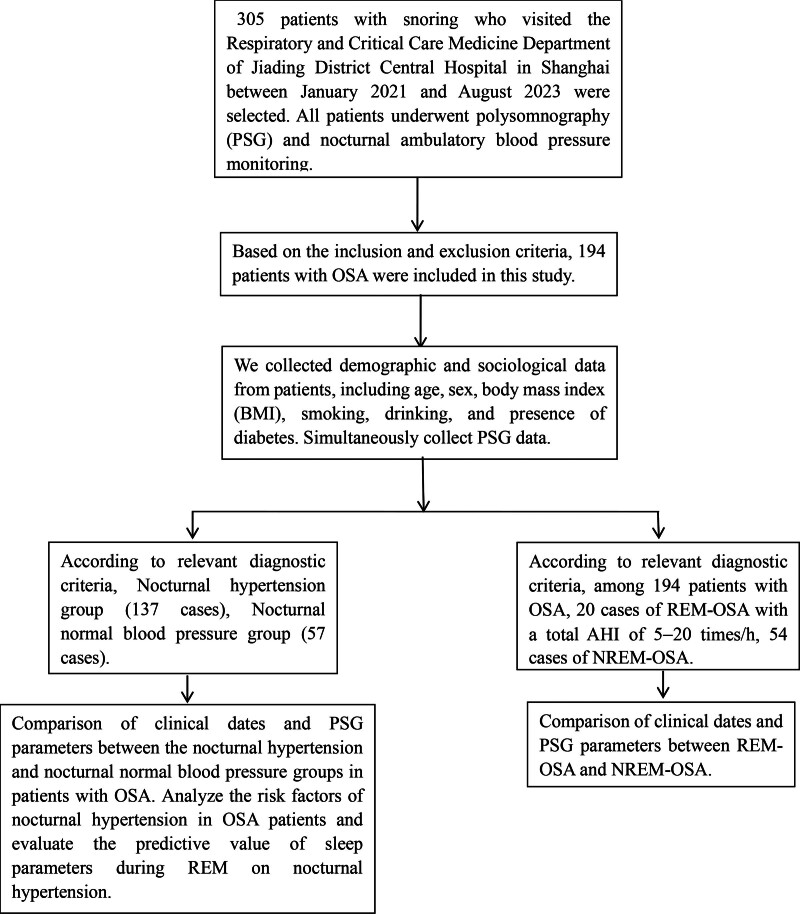
Research flowchart. AHI = apnea–hypopnea index, BMI = body mass index, NREM = nonrapid eye movement, OSA = obstructive sleep apnea, REM = rapid eye movement.

The specific research process is shown in Figure 1.

### 2.5. Diagnostic criteria

The diagnostic criteria were as follows: (1) without the use of antihypertensive drugs, according to the 2013 European Society of Cardiology^[8]^ Hypertension Guidelines and the 2010 China Hypertension Prevention and Treatment Guidelines,^[9]^ nocturnal (sleep) average systolic blood pressure ≥ 120 mm Hg (1 mm Hg = 0.133 kPa) and/or average diastolic blood pressure ≥ 70 mm Hg can be considered nocturnal hypertension. (2) According to the Diagnosis and Treatment Guidelines for OSA developed by the Sleep Respiratory Disorders Group of the Respiratory Disease Branch of the Chinese Medical Association in 2011,^[10]^ the diagnostic criteria for OSA are as follows: patients have nocturnal sleep snoring accompanied by apnea, and physical examination shows stenosis and obstruction in any part of the upper airway. For patients with obvious daytime sleepiness (Epworth Sleepiness Scale score ≥ 9 points), an apnea–hypopnea index (AHI) of ≥5 times/h can be used to diagnose OSA. For patients with insignificant daytime sleepiness (Epworth Sleepiness Scale score < 9 points), AHI ≥ 10 times/h or AHI ≥ 5 times/h and one or more OSA complications, such as cognitive dysfunction, hypertension, diabetes, coronary heart disease, cerebrovascular disease, and insomnia, can also be diagnosed with OSA. (3) REM-OSA was defined as an apnea–hypopnea index (AHI) ≥ 5 times/h, REM-AHI/NREM-AHI ≥ 2, and REM sleep time ≥ 30 minutes. NREM-OSA was defined as AHI ≥ 5 times/h, REM-AHI/NREM-AHI < 2, and REM sleep time ≥ 30 minutes. (4) Smoking history: the patient smoked more than 1 cigarette per day for a continuous or cumulative period of ≥6 months; drinking history: the patient had consumed alcohol at least once a week within the past year.

### 2.6. Research methods

#### 2.6.1. General datas

We collected demographic and sociological data from the patients, including age, sex, body mass index (BMI), smoking, drinking, and presence of diabetes.

#### 2.6.2. PSG and ambulatory blood pressure monitoring datas

A Wanmai SF-A30 multichannel sleep monitor (Hunan Medical Technology Co., Ltd., Liuyang City, China). We performed overnight PSG monitoring of patients, including EEG (4-lead), EOG (2-lead), EMG (1-lead), ECG, oronasal airflow, airflow snoring, blood oxygen saturation, chest movement, abdominal movement, pulse, MIC snoring, posture, body movement, pulse transit time, pulse waveform, respiratory effort, leg movements, and nocturnal ambulatory blood pressure monitoring, using the PSG built-in method of converting blood pressure by monitoring pulse transit time (PTT). The nocturnal average systolic blood pressure, nocturnal average diastolic blood pressure, AHI, REM-AHI, NREM-AHI, oxygen desaturation index (ODI), REM-ODI, NREM-ODI, total sleep time, sleep efficiency percentage, REM sleep time, REM sleep duration as a percentage of the total sleep duration, minimum oxygen saturation, and average heart rate during sleep were also recorded.

### 2.7. Statistical methods

All statistical analyses were performed using SPSS version 26.0 (IBM Corp., Armonk, NY). Metrological data that conformed to a normal distribution are expressed as the mean ± standard deviation. An independent samples *t* test was used to compare 2 groups. Data with non-normal distribution are presented as medians (interquartile ranges). A rank-sum test was performed to compare the 2 groups. Categorical variables were tested using the chi-squared test. Univariate and multivariate binary logistic regressions were used to analyze the correlations between hypertension at night and sex, age, BMI, smoking, drinking, diabetes, AHI, REM-AHI, NREM-AHI, ODI, REM-ODI, NREM-ODI, total sleep time, percentage of sleep efficiency, REM sleep time, percentage of REM sleep time in total sleep time, lowest oxygen saturation, and average heart rate during sleep. We also calculated the odds ratio (OR) and 95% confidence interval (95% CI), and selected independent variables using the forward stepwise regression method. The diagnostic value of the REM-AHI in predicting hypertension in patients with OSA was evaluated using receiver operating characteristic (ROC) curves, and the area under the curve (AUC), maximum Youden index, optimal threshold, sensitivity, and specificity were calculated. In all statistical analyses, statistical significance was set at *P* < .05.

## 3. Results

### 3.1. Comparison of clinical datas between the nocturnal hypertension and nocturnal normal blood pressure groups

Among 194 patients with OSA, there were 137 cases in the nocturnal hypertension group and 57 were in the nocturnal normal blood pressure group. The proportion of males in the hypertension group, BMI, AHI, REM sleep duration, percentage of REM sleep duration in total sleep duration (%), REM-AHI (times/h), NREM-AHI (times/h), ODI (times/h), REM-ODI, NREM-ODI during sleep, and average heart rate at night were higher than that of the normal blood pressure group, ([94.9% vs 77.2%], *P* < .001), ([27.5 ± 4.0] kg/m^2^ vs [26.2 ± 4.0] kg/m^2^, *P* = .035), ([41.4 ± 27.0] times/H vs [28.6 ± 25.7] times/H, *P* = .003), ([123.9 ± 40.2] minutes vs [106.3 ± 44.9] minutes, *P* = .008), ([25.3 ± 7.5]% vs [22.5 ± 8.7]%, *P* = .035), ([36.7 ± 24.1] times/H vs [20.5 ± 21.4] times/H, *P* < .001), ([37.4 ± 26.2] times/H vs [25.9 ± 22.6] times/H, *P* = .004), ([26.6 ± 23.6] times/H vs [18.7 ± 20.6] times/H, *P* = .022), ([22.1 ± 21.4] times/H vs [12.1 ± 14.5] times/H, *P* < .001), ([21.0 ± 18.8] times/H vs [12.2 ± 13.5] times/H, *P* < .001), ([76.6 ± 10.8] times/min vs [69.5 ± 13.4] times/min, *P* < .001). The lowest oxygen saturation in the nocturnal hypertension group was lower than in the nocturnal normal blood pressure group, ([83.9 ± 9.3]% vs [86.4 ± 7.2]% *P* = .043). There were no statistical differences between the 2 groups in terms of age, prevalence of diabetes, smoking history, drinking history, total sleep time, or sleep efficiency (*P* > .05) (Table 1).

**Table 1:** Comparison of clinical dates and PSG parameters between the nocturnal hypertension and nocturnal normal blood pressure groups in patients with OSA.

Characteristics	Nocturnal hypertension group (137 cases)	Nocturnal normal blood pressure group (57 cases)	*P*-value
Male sex proportion	94.9%	77.2%	<.001
Age (years)	40.6 ± 11.4	40.3 ± 14.1	.903
BMI (kg/m^2^)	27.5 ± 4.0	26.2 ± 4.0	.035
Diabetes	35.8%	26.3%	.202
Smoking	39.4%	33.3%	.426
Drinking	38.0%	29.8%	.281
AHI (times/H)	41.4 ± 27.0	28.6 ± 25.7	.003
Total sleep time (minutes)	434.9 ± 89.9	433.9 ± 104.5	.945
Sleep efficiency %	83.4 ± 7.1	83.9 ± 7.7	.689
REM sleep duration (minutes)	123.9 ± 40.2	106.3 ± 44.9	.008
REM sleep duration as a percentage of total sleep duration (%)	25.3 ± 7.5	22.5 ± 8.7	.035
REM-AHI (times/H)	36.7 ± 24.1	20.5 ± 21.4	<.001
NREM-AHI (times/H)	37.4 ± 26.2	25.9 ± 22.6	.004
ODI (times/H)	26.6 ± 23.6	18.7 ± 20.6	.022
REM-ODI (times/H)	22.1 ± 21.4	12.1 ± 14.5	<.001
NREM-ODI (times/H)	21.0 ± 18.8	12.2 ± 13.5	<.001
Minimum oxygen saturation %	83.9 ± 9.3	86.4 ± 7.2	.043
Average heart rate at night during sleep (times/min)	76.6 ± 10.8	69.5 ± 13.4	<.001

AHI = apnea–hypopnea index, BMI = body mass index, NREM = nonrapid eye movement, ODI = oxygen desaturation index, REM = rapid eye movement.

### 3.2. Univariate binary logistic regression analysis of various risk factors for nocturnal hypertension in patients with OSA

Whether patients with OSA had hypertension at night was the dependent variable, and 18 indicators, including sex, age, BMI, diabetes, smoking history, drinking history, total sleep time, sleep efficiency, REM sleep duration, REM sleep duration as a percentage of total sleep duration, AHI, REM-AHI, NREM-AHI, ODI, REM-ODI, NREM-ODI, minimum oxygen saturation, and average heart rate at night during sleep were independent variables. Univariate binary logistic regression analysis was performed separately, and the results showed that 11 indicators (male, BMI, REM sleep duration, percentage of REM sleep duration to total sleep duration, AHI, REM-AHI, NREM-AHI, ODI, REM-ODI, NREM-ODI, and average nighttime heart rate during sleep) were risk factors for nocturnal hypertension in patients with OSA, with crude OR values of 5.487 (95% CI, 2.059–14.625), 1.089 (95% CI:1.005–1.180), 1.01 (95% CI:1.002–1.018), 1.047 (95% CI:1.005–1.091), 1.021 (95% CI:1.007–1.035), 1.035 (95% CI:1.018–1.053), 1.02 (95% CI:1.006–1.035), 1.017 (95% CI:1.002–1.033), 1.033 (95% CI:1.012–1.055), 1.035 (95% CI:1.012–1.058), 1.056 (95% CI:1.024–1.088), all showed statistically significant differences (*P* < .05) (Table 2).

**Table 2:** Univariate binary logistic regression analysis of risk factors related to nocturnal hypertension in patients with OSA.

Characteristics	Crude OR (95% CI)	*P*-value
Male	5.487 (2.059–14.625)	.001
Age (years)	1.002 (0.977–1.028)	.893
BMI (kg/m^2^)	1.089 (1.005–1.180)	.037
Diabetes	1.559 (0.786–3.094)	.204
Smoking	1.301 (0.68–2.489)	.426
Drinking	0.695 (0.358–1.35)	.282
Total sleep time (minutes)	1.000 (0.997–1.003)	.945
Sleep efficiency%	0.991 (0.949–1.035)	.687
REM sleep duration (minutes)	1.01 (1.002–1.018)	.01
REM sleep duration as a percentage of total sleep duration (%)	1.047 (1.005–1.091)	.027
AHI (times/H)	1.021 (1.007–1.035)	.004
REM-AHI (times/H)	1.035 (1.018–1.053)	<.001
NREM-AHI (times/H)	1.02 (1.006–1.035)	.006
ODI (times/H)	1.017 (1.002–1.033)	.032
REM-ODI (times/H)	1.033 (1.012–1.055)	.002
NREM-ODI (times/H)	1.035 (1.012–1.058)	.003
Minimum oxygen saturation %	0.963 (0.925–1.003)	.07
Average heart rate at night during sleep (times/min)	1.056 (1.024–1.088)	<.001

AHI = apnea–hypopnea index, BMI = body mass index, NREM = nonrapid eye movement, ODI = oxygen desaturation index, OR = odds ratio, REM = rapid eye movement.

### 3.3. Multivariate binary logistic regression analysis of risk factors for nocturnal hypertension in patients with OSA

Considering whether the patients had hypertension at night as a dependent variable, excluding collinear independent variables, and taking BMI, age, sex, diabetes, REM-AHI, NREM-AHI, REM-ODI, NREM-ODI, and average heart rate during sleep as independent variables, we performed a multifactor binary logistic regression analysis. The results showed that REM-AHI was an independent risk factor for nocturnal hypertension in patients with OSA (adjusted OR, 1.048; 95% CI, 1.011–1.086; *P* = .01); these results indicated that for patients with OSA, every increase in REM-AHI per hour increases the risk of hypertension by 4.8% (Table 3).

**Table 3:** Multivariate binary logistic regression analysis of risk factors related to nocturnal hypertension in patients with OSA.

Characteristic	Adjusted OR (95% CI)	*P*-value
REM-AHI (times/H)	1.048 (1.011–1.086)	.01

AHI = apnea–hypopnea index, OR = odds ratio, REM = rapid eye movement.

### 3.4. ROC curve analysis of REM-AHI that predicts nocturnal hypertension in patients with OSA

**Figure 2.**
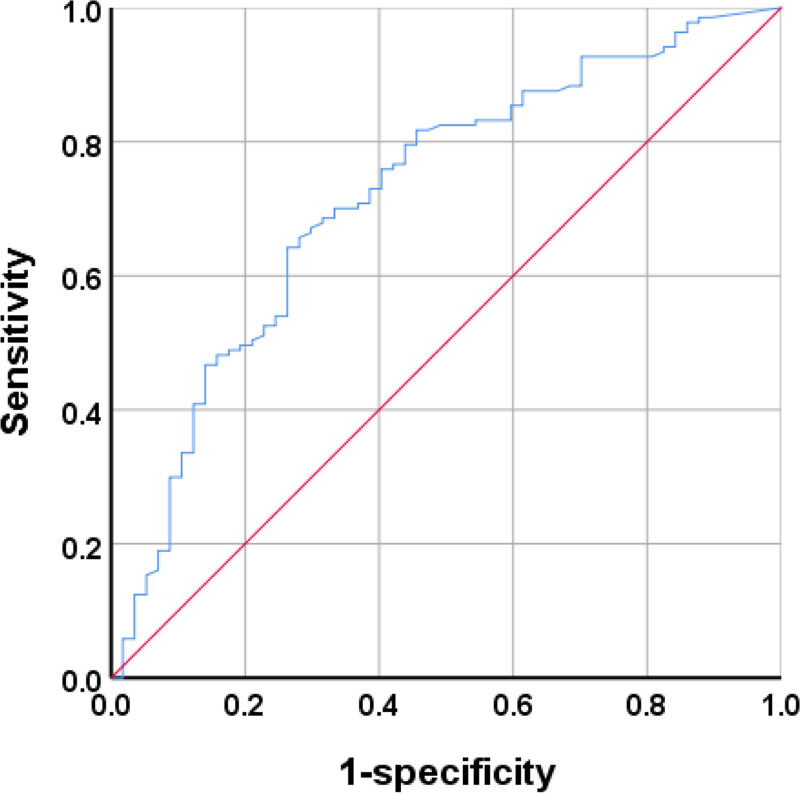
ROC curve of REM-AHI predicting nocturnal hypertension in patients with OSA. AHI = apnea–hypopnea index, OSA = obstructive sleep apnea, REM = rapid eye movement, ROC = receiver operating characteristic.

The REM-AHI had an AUC of 0.721 (95% CI, 0.641–0.801; *P* < .001) for diagnosing nocturnal hypertension in patients with OSA, with a maximum Youden index of 0.379. At this time, the minimum REM-AHI had a critical value, sensitivity, and specificity of 23.6 times/h, 64.2%, and 73.7%, respectively (Fig. 2 and Table 4).

**Table 4:** ROC curve analysis of REM-AHI predicting nocturnal hypertension in patients with OSA.

Characteristic	AUC (95% CI)	Maximum Youden Index	Optimal critical value	Sensitivity %	Specific %
REM-AHI	0.721 (0.641–0.801)	0.379	23.6	64.2	73.7

AUC = area under the curve, AHI = apnea–hypopnea index, REM = rapid eye movement.

### 3.5. Comparison of clinical indicators and PSG parameters between REM-OSA and NREM-OSA

According to relevant diagnostic criteria, among 194 patients with OSA, 20 cases of REM-OSA with a total AHI of 5 to 20 times/h, 54 cases of NREM-OSA, and the proportion of nocturnal hypertension, female sex proportion, and average heart rate at night during sleep in the REM-OSA group were higher than those in the NREM-OSA group (90.0% vs 44.4%, *P* < .001; 50.0% vs 24.1%, *P* = .032; 79.6 ± 13.5 times/min vs 71.3 ± 10.8 times/min, *P* = .008, respectively); the differences were statistically significant (*P* < .05). The onset age in the REM-OSA group was younger than that of the NREM-OSA group (36.9 ± 9.4 years vs 39.0 ± 12.6 years; *P* = .5). Diabetes, smoking, drinking, and AHI in the REM-OSA group were higher than in the NREM-OSA group (30.0% vs 25.9%, *P* = .726; 35.0% vs 29.6%, *P* = .658; 40.0% vs 24.1%, *P* = .177; and 13.3 ± 4.2 times/h vs 12.5 ± 4.5 times/h, *P* = .491, respectively). Oxygen saturation in the REM-OSA group was lower than that in the NREM-OSA group (88.0 ± 4.9% vs 89.2 ± 6.1%, *P* = .429). Although these reference indicators were not statistically significant, they differed between the REM-OSA and the NREM-OSA groups. Larger sample sizes are required to confirm these differences (Table 5).

**Table 5:** Comparison of clinical dates and PSG parameters between REM-OSA and NREM-OSA.

Characteristics	REM-OSA (20 cases)	NREM-OSA (54 cases)	*P*-value
BMI	25.7 ± 4.0	25.8 ± 3.5	.857
Age (years)	36.9 ± 9.4	39.0 ± 12.6	.500
Nocturnal hypertension (%)	90.0	44.4	<.001
Female proportion (%)	50.0	24.1	.032
Diabetes	30.0	25.9	.726
Smoking	35.0	29.6	.658
Drinking	40.0	24.1	.177
AHI (times/H)	13.3 ± 4.2	12.5 ± 4.5	.491
Total sleep time (minutes)	450.0 ± 128.5	445.8 ± 89.8	.895
Sleep efficiency (%)	84.5 ± 6.6	84.2 ± 5.4	.852
Average heart rate at night during sleep (beats/min)	79.6 ± 13.5	71.3 ± 10.8	.008
Minimum oxygen saturation (%)	88.0 ± 4.9	89.2 ± 6.1	.429

AHI = apnea–hypopnea index, BMI = body mass index.

## 4. Discussion

OSA causes partial or complete obstruction of airflow during sleep due to collapse of the upper airways, resulting in hypoxia. Diagnosis is mainly based on monitoring variables, such as oxygen saturation, oronasal airflow, chest abdominal movement, electrocardiogram, and electroencephalogram during sleep using a multichannel sleep monitor. Among them, OSA refers to the disappearance or significant decrease (≥90% decrease from baseline) in oral and nasal airflow during patients’ nighttime sleep, with a duration of ≥10 s. Thoracic and abdominal breathing caused by upper airway obstruction and respiratory arrest persist. However, the respiratory drive function of the central nervous system is normal, and respiratory movement commands continue to be issued to excite the ventilator, resulting in continued chest and abdominal movement. Hypoventilation refers to a decrease of ≥30% in oral and nasal airflow from baseline during sleep, accompanied by a decrease of ≥4% in SaO_2_, lasting for ≥10 s. Alternatively, a decrease of ≥50% in oral and nasal airflow compared with baseline levels is accompanied by a decrease of ≥3% in SaO_2_ for a duration of ≥10 s.^[10]^ Patients with OSA experience increased sympathetic excitability, nocturnal blood pressure, and cardiovascular and cerebrovascular events owing to hypoxemia, frequent arousal, and lack of sleep. OSA is the second leading cause of hypertension,^[11]^ and nocturnal ambulatory blood pressure can better reflect the health status of the population than daytime ambulatory blood pressure.^[12]^ Nocturnal ambulatory blood pressure measurement data are important warning signs for stroke, cardiovascular and cerebrovascular diseases, and coronary heart disease.^[13]^ Therefore, in this study, we collected data on nocturnal ambulatory blood pressure of patients using a blood pressure estimation method based on PTT. Unlike previous cuff methods, PTT is a continuous and short-term process for monitoring blood pressure and does not affect sleep at night. The effectiveness of PTT has been confirmed by comparison with traditional blood pressure measurement methods.^[14,15]^ Table 1 describes the collected demographic and sociological data, PSG, and ambulatory blood pressure data of the 194 patients with OSA (AHI ≥ 5 times/h). A total of 194 patients were assigned to nocturnal hypertension (n = 137) and nocturnal normal blood pressure (n = 57) groups. Clinical and PSG data for the hypertension and normal blood pressure groups were compared, and the number of male patients and BMI in the hypertension group were higher than those in the normal blood pressure group, indicating that male sex and obesity were risk factors for hypertension. Research has confirmed that the prevalence of OSA is 24% to 27% in middle-aged males, 40% to 45% in elderly males, 9% in middle-aged females, and 25% to 30% in elderly females, but exceeds 50% in obese individuals.^[16]^ Owing to the accumulation of fat around the upper airway in obese patients, airway collapse is more likely to occur during nighttime sleep than in patients with a normal BMI. Therefore, obesity is considered an anatomical risk factor for OSA.^[17,18]^ In addition to obesity, other factors can alter the anatomical structure of the upper airways, such as tonsillar hypertrophy, uvular hypertrophy, palatal deformities, and small jaw deformities, which may play important roles in the pathogenesis of OSA.^[19]^ After various causes lead to airway collapse, frequent hypoxemia, microawakefulness, sleep fragmentation, and a surge in sympathetic nervous system activity during nighttime sleep can occur, leading to an increase in blood pressure. In summary, a causal relationship between severe OSA and hypertension was confirmed in animal experiments.^[20–22]^

Which factors in the PSG parameters of OSA patients affect nocturnal hypertension. Considering that there were no statistically significant differences in the total sleep time and sleep efficiency between the nocturnal hypertension and nocturnal normal blood pressure groups, we found that the REM sleep duration, percentage of REM sleep duration to total sleep duration, REM-AHI, REM-ODI, and average nighttime heart rate during sleep were significantly higher in the hypertension group than in the normal blood pressure group (*P* < .05). The lowest oxygen saturation level was significantly lower in the nocturnal hypertension group than in the nocturnal normal blood pressure group (*P* < .05). Therefore, we believe that there is a close relationship between REM respiratory events and nocturnal hypertension. Kleitman and Aserinsky from the University of Chicago in 1953. With the continuous deepening of research on sleep phase, it has been found that REM sleep accounts for approximately 20% of the entire sleep period and is primarily concentrated in the latter half. During REM sleep, the inhibitory effect of the cholinergic-mediated hypoglossal nerve on the genioglossal muscle reaches its maximum, significantly increasing the collapse of the upper airway during this sleep period. However, during NREM sleep, the inhibitory effect on the genioglossal muscle is minimal or even nonexistent.^[5]^ Collapse of the upper airway causes hypoxia during sleep. Hypoxia induces a systemic inflammatory response and oxidative stress, leading to increased endothelin-1 production by vascular endothelial cells, decreased nitric oxide production, and increased peripheral vascular resistance and blood pressure.^[23]^ Meanwhile, periodic hypoxemia and frequent micro-arousal stimulation lead to increased sympathetic excitability, heart rate, cardiac output, and blood pressure.^[24]^ Interestingly, intermittent hypoxia has a stronger effect on increasing sympathetic excitability than persistent hypoxia.^[25,26]^ Our study has shown that the REM sleep duration in patients with nocturnal hypertension is longer than that of patients with nocturnal normal blood pressure, with an average REM sleep duration of 123.9 ± 40.2 minutes vs 106.3 ± 44.9 minutes, *P* < .05, accounting for a percentage of the entire sleep cycle of 25.3 ± 7.5% vs 22.5 ± 8.7%, *P* < .05. The longer the duration of REM sleep, the higher the probability of respiratory events occurring during sleep. For example, the higher the AHI, REM-AHI, and REM-ODI, the lower the oxygen saturation, and the greater the likelihood of nocturnal hypertension. Frequent respiratory events increased the risk of cardiovascular and cerebrovascular disease (Table 1). Findley et al found that the duration of apnea and the frequency and degree of decrease in oxygen saturation during REM sleep in patients with OSA were significantly longer than those during NREM sleep.^[27]^ At the same time, research has confirmed that the frequency and degree of decrease in average oxygen saturation during REM sleep are significantly correlated with the incidence of hypertension.^[28]^ Research using supplemental oxygen has confirmed that asphyxia causes hypoxia and hypercapnia, which stimulates the carotid sinus, weakens vagal nerve tension, increases sympathetic nerve activity, and increases blood pressure.^[29]^

We evaluated the specific impact of these parameters on nocturnal blood pressure in patients with OSA and the diagnostic value of AHI during REM sleep on nocturnal blood pressure using a logistic regression model. Univariate binary logistic regression analysis showed that 11 indicators, including male sex, BMI, REM sleep duration, percentage of REM sleep duration to total sleep duration, AHI, REM-AHI, NREM-AHI, ODI, REM-ODI, NREM-ODI, and average nighttime heart rate during sleep, were risk factors for nocturnal hypertension in patients with OSA (Table 2). After removing the influence of BMI, age, sex, diabetes, NREM-AHI, REM-ODI, NREM-ODI, average heart rate during sleep, and other confounding factors, we concluded that REM-AHI was an independent risk factor for nocturnal hypertension in the OSA population (adjusted OR, 1.048; 95% CI, 1.011–1.086; *P* = .01). For patients with OSA, the risk of hypertension increased by 4.8% every time the REM-AHI increased by 1 time/hour (Table 3). The Wisconsin Sleep Cohort Study found that the REM-AHI was independently related to the incidence rate of hypertension. A REM-AHI ≥ 15 times/h showed a significant threshold effect. In patients with NREM-AHI ≤ 5 times/h, the REM-AHI doubled and the probability of hypertension increased by 24%.^[28]^ The results of the HypnoLaus study showed a significant positive correlation between REM-AHI and systolic and diastolic blood pressure in the REM-AHI > 20 times/h subgroup.^[30]^ The MAIES study found a significant correlation between AHI < 10 times/h and REM-AHI ≥ 20 times/h in male patients and the incidence of hypertension (OR, 2.67; 95% CI, 1.33–5.38).^[31]^ In this study, the ROC curve analysis of REM-AHI to predict nocturnal hypertension in patients with OSA showed an AUC of 0.721 (95% CI, 0.641–0.801; *P* < .001) with a maximum Youden index of 0.379. The optimal critical value of the REM-AHI was 23.6 times/h, with a sensitivity of 64.2% and specificity of 73.7% (Fig. 2 and Table 4).

We concluded that AHI during REM sleep has a significant impact on nocturnal hypertension. This type of hypopnea and apnea, which occur mainly during REM sleep, is referred to as REM-OSA or NREM-OSA. Owing to the lack of consensus among experts on the definition of REM-OSA, the reported prevalence of REM-OSA varies between 10% and 36% in existing data.^[32–35]^ In this study, 194 patients with OSA and AHI of 5 to 20 times/h were selected. Patients with REM-AHI/NREM-AHI ≥ 2 and REM sleep time ≥ 30 minutes were defined as having REM-OSA (N = 20). Patients with REM-AHI/NREM-AHI < 2 and REM sleep time ≥ 30 minutes were defined as having NREM-OSA (N = 54). When comparing clinical and PSG data between the 2 groups, it was found that the proportion of female patients in the REM-OSA group was significantly higher than that in the NREM-OSA group (50.0% vs 24.1%, *P* = .032) (*P* < .05). Furthermore, the age of onset in the REM-OSA group was younger than that of the NREM-OSA group (36.9 ± 9.4 years vs 39.0 ± 12.6 years, *P* = .5). Although the difference was not statistically significant, a significant difference was observed between the REM-OSA and NREM-OSA groups. Larger sample sizes are required to confirm the differences between the 2 groups (Table 5). This was also consistent with a study by Conwell et al^[36]^ who reported that REM-OSA is more common among females and young people. Bahmam et al also found that young age and female sex were independently associated with REM-OSA.^[37]^ In this study, the proportion of patients with nocturnal hypertension was higher in the REM-OSA group than that in the NREM-OSA group. Somers et al^[38]^ found that the average blood pressure was 92 ± 4.5, 116 ± 85, and 127 ± 7 mm Hg during quiet and awake, non-rapid eye movement, and rapid eye movement sleep, respectively. During REM and NREM sleep, blood pressure increases after obstructive events. Meanwhile, in this study, the average heart rate during sleep in the REM-OSA group was significantly higher than that in the NREM-OSA group (79.6 ± 13.5 times/min vs 71.3 ± 10.8 times/min, *P* = .008), with statistical significance (*P* < .05). The oxygen saturation of the REM-OSA group was lower than that of the NREM-OSA group (88.0 ± 4.9% vs 89.2 ± 6.1%, *P* = .429). Owing to the longer duration of apnea and hypopnea during REM sleep, the decrease in oxygen saturation is more pronounced.^[39]^

## 5. Conclusion

AHI during rapid eye movement sleep is an independent risk factor for nocturnal hypertension. For patients with OSA, the hourly increase in the REM-AHI increased the risk of hypertension by 4.8%.

## Author contributions

**Data curation:** Wenjing Zhang, Chunlin Tu, Yanfang Yu.

**Formal analysis:** Wenjing Zhang.

**Funding acquisition:** Chunlin Tu.

**Methodology:** Wenjing Zhang, Chunlin Tu, Yanfang Yu.

**Project administration:** Wenjing Zhang, Chunlin Tu, Yanfang Yu.

**Writing – original draft:** Wenjing Zhang.

**Writing – review & editing:** Yanfang Yu.
